# Biomimetic Approaches in the Development of Optimised 3D Culture Environments for Drug Discovery in Cardiac Disease

**DOI:** 10.3390/biomimetics10040204

**Published:** 2025-03-26

**Authors:** Jenny Shepherd

**Affiliations:** School of Engineering, University of Leicester, Leicester LE1 7RH, UK; js1005@leicester.ac.uk

**Keywords:** drug discovery, cardiac disease, 3D culture, biomimetics

## Abstract

Cardiovascular disease remains the leading cause of death worldwide, yet despite massive investment in drug discovery, the progress of cardiovascular drugs from lab to clinic remains slow. It is a complex, costly pathway from drug discovery to the clinic and failure becomes more expensive as a drug progresses along this pathway. The focus has begun to shift to optimisation of in vitro culture methodologies, not only because these must be undertaken are earlier on in the drug discovery pathway, but also because the principles of the 3Rs have become embedded in national and international legislation and regulation. Numerous studies have shown myocyte cell behaviour to be much more physiologically relevant in 3D culture compared to 2D culture, highlighting the advantages of using 3D-based models, whether microfluidic or otherwise, for preclinical drug screening. This review aims to provide an overview of the challenges in cardiovascular drug discovery, the limitations of traditional routes, and the successes in the field of preclinical models for cardiovascular drug discovery. It focuses on the particular role biomimicry can play, but also the challenges around implementation within commercial drug discovery.

## 1. Introduction

Cardiovascular diseases remain the leading cause of death worldwide, being responsible for approximately 17.9 million deaths each year, 1/3 of these prematurely, before the age of 70 [1,2]. There is massive investment in this area and real success in regard to understanding the basic science which has resulted in a plethora of promising targets for new therapies, yet the progress of cardiovascular drugs to their application in clinics remains slow [3]. The pathway of a drug from discovery to clinic is long, complex, and costly. The likelihood that a new molecular entity entering clinical evaluation will reach the marketplace has been reported to be just 7% for cardiovascular disease, much lower than, for example, oncology [4].

Failure becomes increasingly expensive as a drug progresses along the pathway to the clinic; the earlier we can determine whether a drug is likely to be successful or ultimately fail, the more cost efficient drug discovery becomes. In vitro models will never replace clinical trials, but high-throughput approaches for the rapid screening of potential drug candidates in terms of preliminary efficacy and safety are key. As Kreutzer et al. succinctly state: “The predominant reason for clinical trial failure is lack of efficacy, which hints at limited predictability and transferability of preclinical research to human patients” [5].

There is a tradition of using in vivo models for cardiovascular disease, applied within preclinical testing, with rodent models being particularly common [6,7,8]. These, however, have significant limitations: the species used are fundamentally different, the models used generally lack any long-standing cardiac pathology, and it is rare that concomitant diseases, for example diabetes, are considered [9,10]. The focus has begun to shift towards the optimisation of in vitro culture methodologies, not only because these are undertaken earlier on in the drug discovery pathway, but also because the principles of the 3Rs have become embedded in national and international legislation and regulation. The FDA’s Modernization Act 2.0, overturned the Federal Food, Drug, and Cosmetics Act of 1938, which mandated animal testing for every new drug development protocol [11]. The act advocates for the integration of alternative methods to conventional animal testing, including cell-based assays that employ, for example, human induced pluripotent stem cell (iPSC)-derived organoids, and organ-on-a-chip technologies, in conjunction with sophisticated artificial intelligence (AI) methodologies [12].

In addition, 3D culture has provided a marked improvement over traditional two-dimensional culture, but the standard matrices of Matrigel or other biologically derived gels lack the structural, chemical, and biochemical control needed to mimic specific tissues. Recently, in vitro engineered cardiac tissues have been introduced with the aim of resembling human heart morphology and function and these tissues have been implemented in disease modelling, compound testing, and patient-specific screening [2,13,14,15,16]. There remain, however, significant limitations to the replication of the combination of structural, biomechanical, and biochemical factors. Additionally, cell behaviour is influenced by the local loading environment; native cardiomyocytes experience complex loading, they undergo static and cyclic tension, as well as shear stresses [2]. In order to maximise the relevance of preclinical cardiovascular drug testing, a system is required that provides a complex microenvironment that mimics the native tissue, that provides physiological cues, and that allows for cell–cell and cell–matrix interactions representative of the human heart.

As Khakoo et al. state, much can be learnt from oncology. Here, greater investment in basic research, as well as the application of expedited regulatory pathways, have significantly lowered the barriers to drug development [3]. Oncology research has the advantage that tumour explants are fairly readily available, but for cardiovascular disease, engineered constructs may provide the next best thing. There has been no shortage of advances in the basic science surrounding cardiovascular disease and a plethora of promising targets for new therapies, but there still remains this very significant barrier to development [3].

This review aims to provide an overview of the challenges in cardiovascular drug discovery, the limitations of traditional routes, and the significant successes in the field of preclinical models for cardiovascular drug discovery. It will highlight the particular role biomimicry can play and demonstrate how more representative models can adhere to the 3Rs (refinement, reduction, and replacement) when it comes to animal studies and a reduction in expensive failures at the clinical trial stage.

## 2. Overview of the Drug Discovery Process

**Figure 1:**
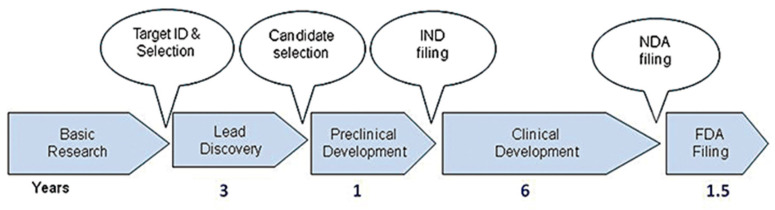
Drug discovery process from target ID and validation through to filing of a compound and the approximate timescale for these processes. FDA, Food and Drug Administration; IND, Investigational New Drug; NDA, New Drug Application. Reproduced with permission from Hughes et al., *British Journal of Pharmacology*, 2011 [17].

Developing a new drug from an original idea to the launch of a finished product is a complex process (summarised in Figure 1), which takes 12–15 years [17]. The process of drug development begins with discovery programmes that result in the synthesis or isolation of compounds that are tested in vitro and in animal studies during preclinical development. Clinical (human) testing then typically proceeds through three successive, sometimes overlapping, phases [18]. A drug discovery programme is initiated essentially due to an unmet clinical need, there is a disease or clinical condition in need of treatment [17]. Simplistically drugs fail in the clinic for two reasons: they do not work, or they are not safe. As Van Norman summarises, approximately 1 in 1000 potential drugs is graduated to human clinical trials after preclinical testing in the United States, and almost 9 out of every 10 new drugs then fails in the human testing phase. These statistics are representative of the developed world [18,19,20]. In a 2016 study, the research and development costs of 106 randomly selected new drugs were obtained from a survey of 10 pharmaceutical firms [18]. The fully capitalised total cost per approved new drug was calculated as USD 2.6 billion far in excess of the oft-reported USD 1 billion [17].

Cardiovascular disease remains the leading cause of death worldwide, yet a number of studies state concerns over the progress of drug development. A 2015 publication, by the Journal of the American College of Cardiology, highlighted real concern over the stagnated investment in cardiovascular drug development [21]. Here, the main reason was given as the high cost of conducting clinical trials in the regulatory environment, the pharmaceutical industry simply did not see the cardiovascular sector as a good return on investment [21]. In 2019, Savoji et al. described investment in drug discovery and drug development as having sky rocketed, but there was still a continued decline in the number of approved drugs [2].

There is a continued need to fill the gap in effective drug discovery and screening. Accurate, predictive models are essential to both study and discover new treatments for cardiovascular pathologies and also to screen for adverse reactions of other pharmaceuticals on the heart [2]. A review of reasons for drug attrition in nonclinical and clinical development showed that cardiovascular toxicity occurred more frequently than hepatotoxicity when serious adverse reactions and withdrawal of drugs from the marketplace were analysed [22]. Whilst it has been reported that 70% of human toxicity can be approximated during the preclinical stage, there are other subtler and higher risk cardiovascular events that may only be observed when drugs are administered to humans for longer periods of time and in larger populations [23].

A basic understanding of the pathophysiology of cardiac disease is necessary in order to understand the requirements of predictive models; this, along with the current shortfalls in cardiovascular disease focused drug development, must provide the key impetus for future developments in preclinical testing. In the following section of this review, brief overviews are provided of the pathophysiology of cardiac diseases, with particular focus on the limitations of current drug discovery. This is then followed by a more indepth discussion of preclinical cardiovascular drug testing specifically.

## 3. Pathophysiology of Cardiac Disease

This review focuses on drug discovery for diseases of cardiac tissue specifically rather than the vasculature, thus the focus is on the pathophysiology of cardiac diseases, such as arrhythmia and cardiomyopathy. However, it cannot go unsaid that atherosclerosis and coronary heart disease still remain the two conditions with the highest instances of CVD hospital admissions in the UK, reported as 25.8% of all CVD hospital admissions in 2019 [24]. Atherosclerosis has also been described by Scott as the driver of the emerging epidemic of heart failure [25]; hence, it will be described prior to the consideration of arrhythmias, heart failure, and cardiomyopathies.

### 3.1. Atherosclerosis

Atherosclerosis is a chronic inflammatory disease characterised by the thickening, hardening, and narrowing of the arteries, due to deposits of cholesterol, fatty deposits, calcium, and fibrins [26,27]. Atherosclerosis is the primary cause of coronary heart disease (CHD) and stroke, the first and third most prevalent causes of death in the UK, respectively [24]. On average, 68,000 deaths are caused by CHDs and over 30,000 deaths are caused by strokes in the UK alone [24]. Atherosclerotic lesions develop over a lifetime and are largely irreversible; therefore, clinically the most important goals are prevention and early diagnosis [28].

During atherosclerosis, cholesterol deposits accumulate in large and medium arteries, leading to the proliferation of macrophages and smooth muscle cells within the arterial wall. The enlargement of atherosclerotic plaque gradually impinges on blood vessels and blocks blood flow. Atherosclerosis can also result in thrombosis, which may ultimately lead to myocardial infarction [2]. This is where the oxygen supply to myocytes becomes restricted. Myocyte contraction requires oxygen and limited oxygen supply following infarction results in cardiac damage and cell death [2,29].

Recently there have been significant advancements in the understanding of molecular and cellular interactions in atherosclerosis; there is a greater understanding of the links between genetic and environmental factors and real advances in both diagnostics and therapeutics [28]. With the awareness of the importance of the genetic profiles of patients with cardiovascular disease, comes the possibility of tailored treatment; however, this additionally requires tailored preclinical drug discovery and alternative methodologies to the traditional well-plate culture technique.

As Chen et al. state, in vitro models can provide real opportunities to obtain more data, more efficiently and economically, compared to animal models [30]. In vivo atherosclerosis models suffer due to interspecies differences in the genome, limited genetic variability, and low throughput. There have been advances in 2D culture, such as cell sheet tissue engineering and microfluidic technology, that have greatly improved its physiological relevance; however, 2D cultures are still far from being able to mimic human pathophysiology [30]. Again, it should be emphasised that an ideal in vitro system should mimic the 3D human tissue architecture with proper cellular components and disease features; however, with 3D culture comes inevitable challenges in regard to the cell number and viability, as well as imaging and characterisation challenges.

Table 1, modified from Chen et al., provides an excellent overview of the various in vitro models implemented in atherosclerosis research, with their various advantages and key challenges. Greater detail on these methods will be provided in the Section 5 of this review, but with more of a focus on cardiac (rather than vascular) applications.

### 3.2. Heart Failure

**Figure 2:**
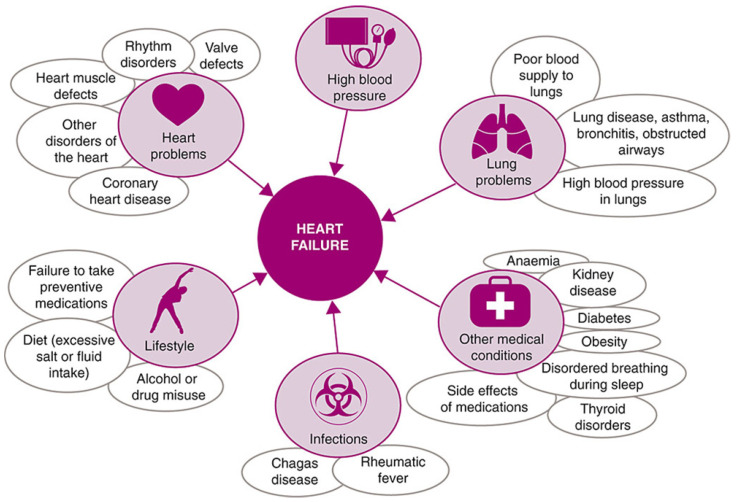
Common causes of heart failure, reproduced under the Creative Commons CC BY Licence from Ponikowski et al., *ESC Heart Failure Volume 1* (2014) [32].

Heart failure describes a range of conditions, whereby the heart is unable to pump enough blood to the rest of the body. It is the general term given to the chronic stage of any disease leading to cardiac functional impairment [31] and has a diverse aetiology, as shown in Figure 2.

In economically developed countries, up to one in five people is expected to develop heart failure at some point in their life [32]. The heart is a remarkably adaptable organ; if demands on it are increased, it is able to remodel itself, maintaining its output, at least in the short term. Longer term, however, the heart’s pumping activity may decrease, and the valves may malfunction as the heart enlarges, dilates, and stiffens. Eventually, these changes manifest as overt symptoms of heart failure [32].

Not surprisingly with such stark statistics concerning the incidence of heart failure, there have been extensive cellular, molecular biological, and biochemical studies on heart failure. Unfortunately, however, the development of successful heart failure treatments has been disproportionately low and, throughout the last two decades, relatively slow progress has been made [33,34]. Traditional treatments based on neurohumoral and hemodynamic modulation quickly reached a therapeutic plateau, with little additional advantage garnered from increased doses or alternative drugs targeting the same pathways [35]. Correale et al. suggest this is due to the non-targeted and non-personalised nature of these approaches, as they are not based on individual pathophysiology, also because the effect of comorbidities are not considered [35]. They report that these comorbidities result in a systemic proinflammatory state, which causes coronary microvascular endothelial inflammation. As such, there are additional targets that should be considered, namely the influence of drugs on cardiomyocytes and the myocardial interstitium and functional performance, such as microcirculation and inflammation. Essentially, it is insufficient to consider just one single target. The inter-connected nature of heart failure and other comorbidities, as well as the numerous postulate mechanisms of failure, present real challenges in regard to any sort of predictive capabilities for the in vitro analyses of drugs for heart failure.

Key drug studies for heart failure involve animal models, but as Janssen et al. report, the data generated by these drug studies are often heavily impacted by the choice of species and how relevant the conditions are to the physiological disease state [34]. Their claim is that approaches that use multiple models and that are not just restricted to small rodents but involve verification withboth larger animal models and viable human myocardium are needed to advance drug discovery for the very large patient population that suffers from heart failure [34]. This is clearly costly, but also contrary to the desires of the ‘3Rs’, when it comes to animal models in medicine. Advancements in 3D culture methodologies are to key to any possible replacement of animal models with either in vitro or ex vivo analyses.

### 3.3. Arrhythmia

Normal heart function relies upon cardiac action potentials that cause the coordinated contraction of cardiomyocytes (CMs), which pump the blood forward to peripheral tissues [2,36,37]. The potential is the result of a series of highly ordered openings and channels in the CM membrane, conducting the AP signal from one CM to the next [2,38]. Differential expression of the channels in CMs in various regions of the heart results in unidirectional electrical waves. Disruption of this ordered wave, through physical obstacles, such as dead or ischemic tissues, fibrosis, and inflammation, can cause arrhythmia [39]. Arrythmia can also be caused by imbalanced channel activity and physical and phenotypic cell changes [2,36]. Atrial fibrillation is the most common cardiac arrhythmia accounting for around 80% of the total cases. Whilst there have been significant developments in surgical procedures for atrial fibrillation, such as catheter ablation, treatment is overwhelmingly through antiarrhythmic drug therapy to restore and maintain sinus rhythm [40]. There remains a desire to develop safe and effective drugs that can rapidly cardiovert AF. Drugs that have shown significant potential in Chemical cardioversion, such as Vernakalant, have been approved for use in Europe, but have not been approved by the FDA. Reported, as a consequence of a death during a random clinical trial, there have also been significant adverse events attributed to Vernakalant in case reports, such as hypotension, atrial flutter, or atrial tachycardia [40].

It has been suggested that whilst the primary focus in pharmacological AF therapy has been the development of antiarrhythmic drugs, with increasing awareness of the progression of the disease, there has been a shift of focus to drugs that target AF-related atrial electrical and structural modelling [41]. This focus means target analysis and preclinical testing through analysis of sodium channels and electrophysiology in simple 2D culture may no longer be effective. Here, preclinical studies should involve replication of the native tissue environment (ideally in the diseased state), in combination with electrical stimulation and monitoring, requiring a much more biomimetic approach to preclinical analysis.

### 3.4. Cardiomyopathy

Cardiomyopathy is a general term encompassing a variety of symptoms, including heart muscle enlargement, thickening, and rigidity. Cardiomyopathy has various phenotypes [42,43,44], which are beyond the scope of this review, some of which have known causes, others much less so, but they generally result in weakening of the heart and modification of its ejection fraction [2].

Cardiac muscle is the functional unit of the heart; it is involved in force generation and propagation of electrical signals through cardiomyocyte-rich tissue, resulting in rhythmic pump contractions [15]. It is composed of various cell types, the most abundant being CMs, pericytes, endothelial cells, and fibroblasts, and the proportion of each cell type in the heart remains a significant topic of discussion [15]. Complexity is further added by the variation in cellular composition and transcriptional signals across the heart, which give the specific anatomical regions their highly specialised functions. Tenreiro et al. discuss this in the context of the elevated challenges of engineering artificial cardiac tissue, but the same challenges exist in the development of preclinical drug analyses and the creation of relevant systems of culture [15].

## 4. Cardiac Tissue Structure

Traditionally, the myocardium has been viewed as a purely muscular organ, with Braunwald describing the myocardium as being composed of individual striated muscle cells, 10–15 mm in diameter and 30–60 mm in length [45]. Each fibre contains multiple cross-banded strands (myofibrils), which run the length of the fibre, and are composed of a serially repeating structure, the sarcomere [45]. However, this is a simplistic view. The extracellular matrix of the myocardium is structurally elaborate, largely consisting of fibrillar collagen types I and III. Myocardial fibres in native tissue (predominantly collagen type I) have been shown to be aligned in layers, with their orientation varying transmurally [46], with myocardiocyte alignment mirroring the fibre orientations [47].

This extracellular matrix is responsible for the support and alignment of myocytes and capillaries and is a major determinant of myocardial tissue stiffness [48]. If disproportionate accumulation of collagen occurs through fibrosis, then heart tissue stiffness increases; a degradation of collagen, on the other hand, can result in a reduction in stiffness, which can lead to chamber dilation, wall thinning, and even rupture of the myocardium [49].

Golob et al. considered heart disease from an engineering perspective, treating it as a reduction in biomaterial performance [50]. In materials science, performance is a direct result of structure, properties, and processing. The same considerations apply to ventricular function, but with significantly increased complexity compared to the average engineering material. Ventricular function, often clinically assessed using ejection fraction, is dependent upon the performance of the sarcomere (the basic contractile unit of muscle fibre), loading condition, and interventricular interactions, as well as action potential conduction [50]. The arrangement of intra and extracellular proteins, as well as their isoforms, also contributes to the material properties of ventricular walls [50]. The right ventricle is embryonically, structurally, and functionally distinct from the left ventricle [50]. Protein isoforms affect the material properties and some isoforms shift during heart failure. Different isoforms of various proteins may interact during various phases of the cardiac cycle [50]. It is this complexity in cardiac tissue structure that results in the significant challenges faced when replicating the cardiac environment for representative preclinical testing.

## 5. Preclinical Testing in Cardiac Drug Discovery

The aim of preclinical studies is to demonstrate a proof of principle, that a drug is efficacious, with minimal side effects. Only once this proof is established, is a drug then eligible for clinical testing [51]. Preclinical studies generally consider the pharmacodynamic, pharmacokinetic, and toxicologic properties of compounds in order to predict adverse outcomes, define safety windows, and estimate dose ranges to support and design subsequent clinical trials [5].

Traditionally high-throughput in vitro 2D assays are typically followed by in vivo models, prior to the first in-person studies. Failure of a drug becomes increasingly expensive as you move along the drug development pathway; the earlier a drug is identified as likely to be successful or ultimately fail, the more cost efficient drug discovery becomes. 2D monocultures are inherently unable to represent the complexity of in vivo cardiac structures, dynamics, and microenvironment [52]; thus, drugs might appear promising and progress further down the pathway, only to fail at later stages, when costs are much greater.

The particular challenge of in vitro analysis in cardiac disease modelling is that diseases tend to be complex, multi-step cellular and molecular processes that develop over weeks and months in many cases, with numerous other comorbidities [53]. The cardiovascular system is hugely complex and shows a broad interdependence with circulation, blood vessels, and blood constituents, as well as the nervous and renal systems; all of this, makes it especially challenging to model [2].

**Figure 3:**
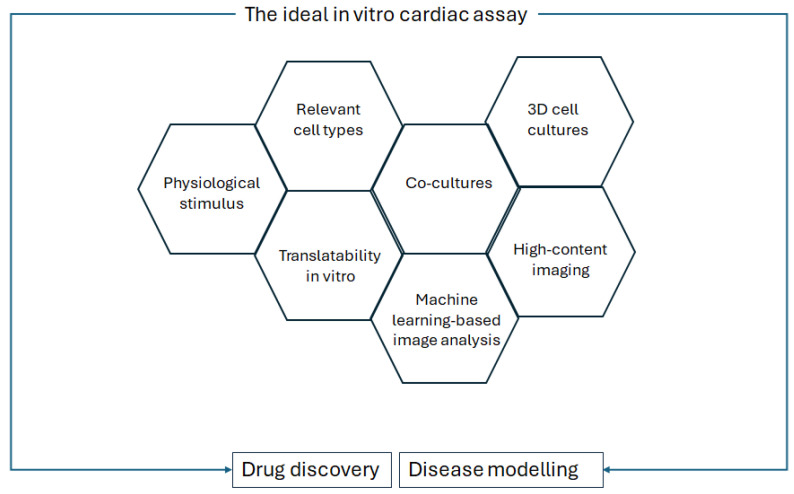
Requirements of the ideal in vitro cardiac assay for drug discovery and disease modelling, modified from Palano et al., *Frontiers in Physiology*, 2021 [53].

In Palano et al.’s review on in vitro assays for drug discovery for cardiac fibrosis, the challenges to recapitulating cardiac fibrosis in a dish are described, as well as the features that should be taken into account for optimised in vitro culture [53]. These features are replicated for a general in vitro cardiac analysis, as shown in Figure 3.

Even if a single cell type can be considered representative, multiple biomarkers are typically required in order to provide the necessary information in a drug-screening cascade. More physiologically relevant cell models often come with higher complexity and, thus, lower throughput for drug screening [53].

### 5.1. Cell Selection in Cardiac Drug Testing

**Figure 4:**
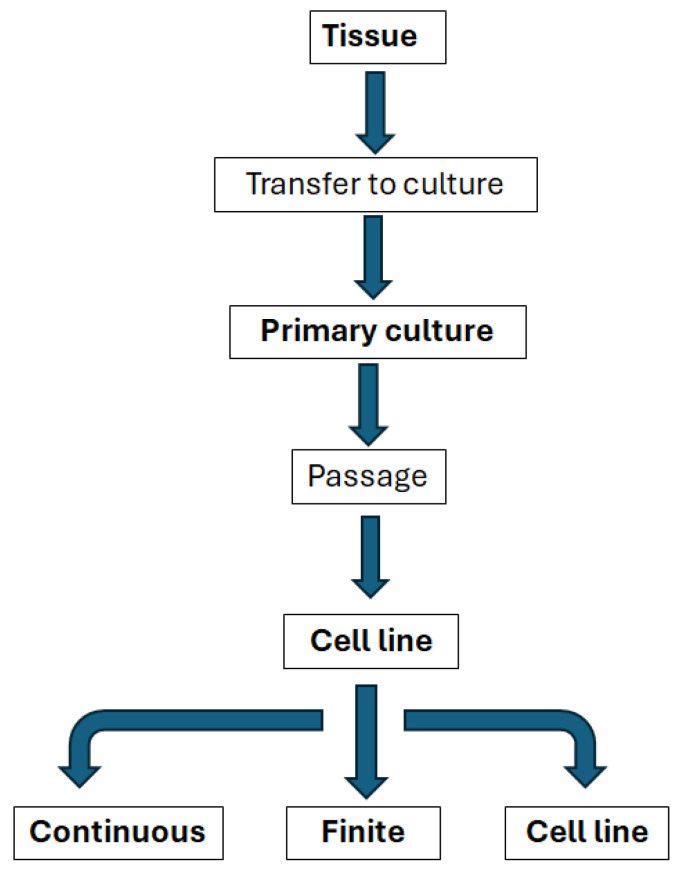
Relationships between the main types of in vitro systems, modified from Coecke et al., *Alternatives to Laboratory Animals*, Vol. 33 (2005) [54].

The selection of the appropriate cardiac cell source is essential for any attempt to engineer an in vitro environment. It is critical that the cells can retain their phenotype and resemble native heart tissue without losing their biological functions [2]. Standardisation of in vitro analysis is key [54] and this requires an understanding of how cell types may be classified (Figure 4).

Isolated organs and tissues taken for direct use from human or animal donors have a range of potential in vitro applications, but are often difficult to standardise and have complex environmental and nutritional requirements, interdonor variation not to mention significant limitations to supply, particularly for cardiac applications [54].

#### 5.1.1. Primary Cells

Primary cells are directly harvested from tissue; they have a limited lifespan but, typically, retain similar qualities to their in vivo phenotype. There are particular challenges with primary human cardiomyocytes, as they can only be isolated from small cardiac biopsies, which are rarely taken, and they have an extremely limited ability to proliferate [2,55,56]. There is also the added challenge that adult cardiomyocytes de-differentiate and do not survive medium-term culture; culture generally utilises neonatal cardiomyocytes and they demonstrate significant differences in structure and function to mature cardiomyocytes [13].

#### 5.1.2. Cell Lines

Standard in vitro models often use cell lines due to their availability and ability to undergo expansion [54]. Cell lines are able to multiply for extended periods in vitro and are, therefore, able to be maintained through serial subcultures [54]. Cell lines can be further broken down into finite cell lines that may replicate for up to 60–70 cell doublings, but will eventually reach a state of senescence [54]. These cell lines still have a useful lifespan in vitro and have been maintained as well-characterised and quality-controlled cell banks. Many finite cell lines show good genetic stability [54]. Whilst continuous cell lines do not reach this state of senescence, they may well undergo substantial and irreversible changes and, therefore, need to be carefully characterised; they are typically derived from tumours or normal embryonic tissues [54].

Because of the significant challenges associated with both isolating and maintaining primary cells from cardiac muscle tissue, it is incredibly challenging to generate cell lines (see the flowchart in Figure 4). There are cell lines that are used occasionally incardiovascular drug discovery, HL-1, for example, is a cardiac muscle cell line derived from the AT-1 mouse atrial cardiomyocyte tumour lineage. These cells have the same morphology as differentiated cardiomyocytes, maintain the biochemical and electrophysiological properties and the ability to contract, while they can be passaged several times [57].

Stem cell lines are continuous cell lines that maintain the ability to differentiate into diverse cell types; they require real care in maintenance, handling, and preservation to retain their stem cell characteristics.

### 5.2. The Role of Induced Pluripotent Stem Cells (iPSCs) in Cardiac Drug Discovery

The first iPSCs were generated in 2006 by Takahashi and Yamanaka. Pluripotent stem cells were induced from adult murine fibroblasts through the introduction of just four factors under embryonic stem cell culture conditions [58]. Since then, they have been widely established as a research platform for determining disease mechanisms [59,60,61,62,63] and in drug discovery research [64,65,66].

It has been claimed that iPSC technology has triggered a paradigm shift in drug discovery and clinical trial landscapes, with the claim that it circumvents issues posed by animal studies and primary cells, enabling mass production of disease and patient-specific functional somatic cells [51]. iPSCs have also been described as a platform to effectively study a range of patient- and disease-specific heart disease conditions in vitro [51], including Long QT syndromes [67], hypertrophic cardiomyopathy [68], and various arrhythmias [69,70]. They have also proven useful in patient-specific drug screening and drug toxicity testing [51,71,72,73]. The use of HiPSCs has achieved regulatory recognition, with both the FDA and EMA approving them for nonclinical cardiac safety risk assessments, and they have additionally been included in the FDA Modernization Act for testing drug effectiveness [74].

Dronedarone is an example of a drug that was touted as a real game changer in the maintenance of sinus rhythm in patients with atrial fibrillation, yet ultimately it was found to result in heart failure and chronic atrial fibrillation. Paik et al. claim that preclinical testing with iPSC-CMs and cardiac organoids could have averted the resulting financial catastrophe and saved lives [51].

As Savoji et al. summarise, recent advances in iPSC technology have addressed limitations around low reprogramming efficiency, lengthy differentiation processes, high cost, and variability among iPSC lines, enabling the use of these cells for in vitro disease platforms [2]. iPSCs derived from patients with Barth syndrome (BTHS) were successfully differentiated to CMs to develop an in vitro microfabricated disease platform to model cardiomyopathy in patients with BTHS [75].

There are challenges in the use of iPSC-derived cardiomyocytes, not least concerning the generation of adult-like and tissue-specific subtypes of iPSC-derived cells. In addition, iPSC-derived cardiomyocytes are generally immature in nature, lacking organised myofibrils, being much smaller than mature CMs, and generating a much weaker contractile force [76].

In order to determine the maturity of CMs, it is possible to consider cellular self-organisation, calcium transients, contractility, and electrical activity [11], but these methodologies are not rigorous nor are they always quantifiable. Differential characteristics between immature and mature CMs have also been reported using RT-qPCR, immunofluorescence, and proteomic analysis [16,77]. Topographical cues, biochemical stimuli, and the development of 3D-engineered models all play a role in iPSC-CM maturity [10]. Future studies addressing HiPSC-CM immaturity are absolutely essential for in vitro drug development; their lack of maturity has been claimed to limit their current utility [10].

Because cardiovascular diseases affect distinct chambers of the heart, it is necessary to develop methods to generate chamber specific iPSC-CM cellular subtypes. Chamber-specific CMs are functionally distinct, for example, exhibiting different calcium handling properties. This has been achieved using modulated retinoic acid signalling during cardiomyocyte differentiation [78]. Other biomimetic approaches and tissue engineering techniques have been shown to enable the creation of electrophysiologically distinct atrial and ventricular 3D microtissues, with chamber-specific gene expression and drug responses [79].

Whilst there has been very significant focus on the differentiation of iPSCs to mature CMs, this single cell type is far from sufficient for predictive preclinical testing. Additionally, iPSCs must be differentiated into other cell types relevant for various cardiovascular diseases, for example endothelium, smooth muscle, fibroblasts, and even hematopoietic lineage cell types, such as monocytes and lymphocytes [80].

The use of iPSCs removes the ethical concerns around the use of embryonic stem cells; they also retain the genetic information and provide a real opportunity to advance personalised medicine [51]. However, they are not yet suitable for high-throughput screening and remain prohibitively expensive. As Paik et al. describe, the cost of personalised drug discovery using iPSC platforms could be enormous, as each person would require a blood or tissue sample to be reprogrammed and subsequently differentiated, a process that can take up to 6 months for each individual. Further, the multifaceted pathophysiology of cardiovascular diseases involving multiple cell types in a number of organ systems, mean in vivo model validation is still typically required [51].

### 5.3. In Vivo Models

Much of the understanding of the aetiology, pathogenesis, pathophysiology, progressions, and underlying mechanisms of cardiovascular diseases have been garnered by in vivo models [81,82,83,84,85,86]; they provide valuable tools in drug discovery and play a fundamental role in validating and delving deeper into observations made in various in vitro cell models [81]. The choice of an appropriate animal model is a challenging process and there is a real emphasis on the scientific community to reduce animal use from animal welfare and research ethics standpoints, and to comply with the 3Rs (replacement, reduction, and refinement) [2].

Small animal models, particularly mice and zebrafish, have become invaluable tools for understanding pathophysiology and testing potential therapies. As Risato et al. summarise in the context of arrhythmic cardiomyopathy, mice models cannot fully replicate the complexity of the human condition, but do provide valuable insights into gene involvement, signalling pathways, and disease progression [81]. Approximately 99% of human genes have direct orthologues in mice and there are notable parallels in morphology, cell biology, and physiology. Rodent studies have constraints around cost, time, and ethics and these can be addressed with theuse of zebrafish, which also have genetic advantages [81]. Zebrafish share orthologs for approximately 71% of human proteins and 82% of which are linked to human diseases [87]. Particularly for cardiovascular disease, the structural and electrical similarities between zebrafish and the human heart make zebrafish an effective model for understanding the cardiac genes associated with cardiovascular disease [88].

Models of heart disease in small animals, particularly rats, have been useful for the assessment of pharmacological therapies. Several target genes have also been identified in genetically modified mouse models. These genes have proven to be essential in the initiation and progression of heart disease [89]. There are a range of methods for inducing heart disease; for example, treatment of rats with isoproterenol has been used in order to induce cardiac hypertrophy, myocyte necrosis, and interstitial cell fibrosis [82,90]. Less reproducible is the electrical method, wherein a soldering iron is applied to the epicardium of the left ventricle [91].

Whilst there are many advantages of working with mice (ease of handling, short pregnancy times, etc.), there are two important limitations: the small size of the heart and structural differences compared to the human cardiovascular system. However, the major advantages lie in the availability of transgenic and knock-out strains and the relative ease with which new genetic modifications can be introduced [89].

Rodent and human hearts differ in their architecture, heart rates, oxygen composition, contractility, protein expression, and stem cell populations, so there is a need for models of heart failure in large animals [89]. The first large animals considered were dogs, but pigs are more typically considered the preferred model [89]. The similar size and cardiac physiology of pigs and humans mean that this model offers major advantages over models involving other species. However, the method requires specialised equipment, dedicated surgical facilities, and skilled personnel, limiting the number of laboratories able to conduct these studies [89]. Table 2 highlights the major advantages and disadvantages of small and large animal models of cardiovascular disease [2].

## 6. Biomimetics in Cardiac Drug Discovery

Tissue engineering and regenerative medicine is a broad research field aimed at providing regenerative alternatives to either synthetic replacement tissue or harvested tissue for transplantation. Tissue engineering requires the triumvirate of supporting scaffold, cells, and biological factors [92], and it is beneficial for the scaffold to mimic certain advantageous characteristics of the natural extracellular matrix; these characteristics may be compositional, structural, mechanical, or inclusion of biologically functional moieties [93].

As Grayson et al. state, tissue development in living organisms is orchestrated by cascades of regulatory factors interacting at multiple levels in space and time [94]. The same is equally true of disease progression or indeed drug response. Whole animal models provide ‘biologic fidelity’ (at least for a given species), but offer limited control over the specific environment and limited real-time insight. Traditional cell culture provides control over the cellular environment and precise insight into cellular processes, but it is ultimately an oversimplified 2D experimental context [94]. First, analyses of the molecular modes of action and cellular toxicity are usually performed and will probably still remain in two-dimensional (2D) cell cultures for the foreseeable future. However, as stated above, these simple systems are unable to replicate the relevant systemic effects in in vivo conditions, thereby limiting their significance for the determination of drug safety [5]. Throughout history, the key outcomes of in vivo dose–responses for human risk assessments, systemic effects, interactions between tissues and organs, specific organ sensitivity, chronic effects, and the pharmacokinetic profile of an Investigational New Drug have relied upon in vivo models, but this is changing with more physiologically relevant cell types and more representative matrices for cultures.

Numerous studies have shown myocyte cell behaviour to be much more physiologically relevant in 3D cultures compared to 2D cultures [95,96,97], highlighting the advantages of using 3D-based models for preclinical drug screening [98]. Biochemical, gene expression, or single-cell assay high-throughput drug screening (HTS) technologies have been highly successful in developing selective and reliable assays of compounds in a rapid and economical manner; they have been able to screen millions of compounds to date [98]. However, the approach is the subject of considerable criticism, as the methods rely on “one-gene, one-protein, one-target”, they do not take into account a drug’s effects on multiple intracellular messenger pathways or the general complexity of 3D tissue [98].

In the clinical scenario, a changing 3D morphology gives rise to mechanical stimuli that act in combination with biological factors to regulate tissue development, maturation, and remodelling [94]. In the case of cardiac tissue, the propagation of electrical signals across specialised intracellular junctions produce mechanical contractions that pump blood forward. Coupling between electrical pacing signals and macroscopic contractions is crucial for heart development and function and, thus, must play a significant role in the analysis of cell behaviour during pathology and drug treatment [94]. The synchronisation of a constant mechanical load, electrophysiology, and the supply of sufficient nutrients and oxygen is complex. Electrical stimulation in vitro has been identified to guide cellular alignment, coupling, and contractile function, key requirements that represent the physiological conditions and, thus, are important in predictive models for drug testing [5].

As Nam et al. state, adequate recreation of an in vivo environment in controlled in vitro conditions is accomplished through the careful modulation of chemical and mechanical inputs within a designed culture platform; the correct physical and chemical micro-cues affect the ability of cells to grow, proliferate, differentiate, and mature. A scaffold helps recreate the physical in vivo microenvironment and enables the cells to grow, with appropriate morphologies [98]. This combination of a physiologically relevant cell population, extracellular matrix composition and mechanics, and physical stimulations must all be considered in full for a truly biomimetic approach to environments for drug discovery [99].

### 6.1. Microfluidics

As has been stated already, standard static well culture approaches do not capture the complexity of the in vivo environment, yet it is desirable not to lose the high-throughput nature of these approaches. In vitro models lack a physiologically relevant co-culture of myocardial cells, a 3D biomimetic environment, and may use non-human cells [100]. In vivo models provide a dynamic environment, constantly perfused with blood and cells that are continually stimulated by chemical, mechanical, and electrical cues. Animal and human cell responses are, however, in many, cases significantly different [101]. Specifically for cardiac drug development, animal models have not sufficiently mimicked the human myocardium, as is evident by the very low clinical translation of cardiovascular drugs [100]. Recently, there has been a wealth of literature considering so-called ‘organs on a chip’, novel platforms merging advances in microfluidics with microfabrication. Microscale platforms enable the precise delivery of fluids, reduced reagent volumes, and high-throughput screening [101,102]; they also enhance reproducibility.

There are two general categories of microfluidic devices; the simplest merely detect cells or markers, whilst the more advanced are designed to study the physiology of disease and drug response in situ. The latter, requires the culture of tissues or ‘organs’ on a chip to establish models in order to understand the complex relationships between cells and the microenvironment used in order to screen drugs [103]. There is contention around whether microfluidics can be considered biomimetic or even truly 3D culture; however, as Khanna et al. succinctly state, “Cardiovascular organ-on-a-chip (OoC) devices are composed of engineered or native functional tissues that are cultured under controlled microenvironments inside micro-chips. These systems employ microfabrication and tissue engineering techniques to recapitulate human physiology” [104].

**Figure 5:**
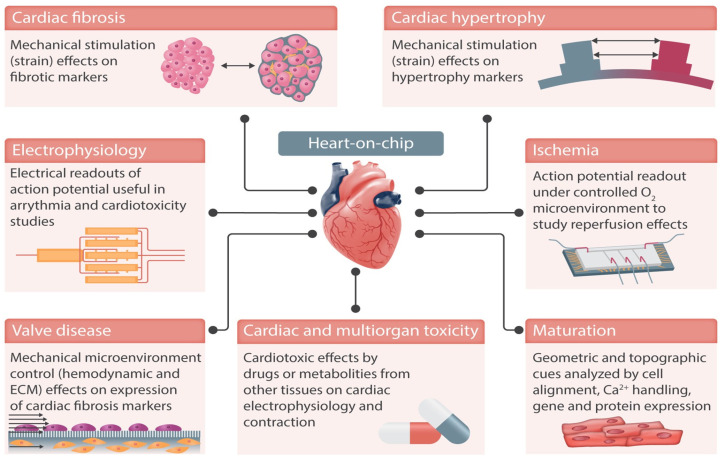
Heart-on-chip devices can replicate cardiac functions in vitro and integrate sensing units to monitor the cells in culture, e.g., action potential. Examples of cardiovascular diseases that can be replicated in these devices include ischaemia and cardiac fibrosis. Integrated electrodes and mechanical actuation enable monitoring and stimulation of cells in culture, better replicating the cardiac microenvironment. Reproduced from Paloschi et al., *Cardiovascular Research*, 2021. 117(14): p. 2742–2754 [105] under the Creative Commons CC BY Licence.

Figure 5, reproduced from Paloschi et al. [105], demonstrates some of the possible applications of heart-on-chip technologies. These technologies are designed to mimic the dynamic conditions of the cardiovascular system, paying particular attention to structural organisation, shear stress, transmural pressure, mechanical stretching, and electrical stimulation [106].

Microfluidic models help to understand the causes of disease progression, as well as providing insight to treatments. Veldhuizen et al., for example, employed human pluripotent stem cell-derived cardiomyocytes, in combination with a microfluidic platform and co-culture with interstitial cells. The specific surface topography of the fluid channel mimicked the anisotropic structure of the human myocardium [107]. The tissue exhibited an enhanced mature cellular structure, protein expression, gene expression, and tissue function.

A ‘myocardium on a chip’ has also been developed, using 3D cell-laden hydrogel constructs and HiPSC-derived myocardial cells, in combination with HiPSC endothelial cells and physiologically relevant shear stresses [100]. The creation of heart constructs on microfluidic platforms have been considered combining bioprinting, enabling the combination of topological complexity and multiple cells and materials [108]. However, as Ellis states, the challenge posed when interfacing bioprinted heart models with vascular models for controlled perfusion remains [100].

For the realisation of reliable heart-on-chip models, cell development should be supported by a mature electrophysiological conduction system, such as synchronised beating [100]. Custom systems have been developed with integrated electrodes on the chip, for example, Zhang et al.’s chip with a platinum-based 3D pillar electrode platform, with a cell growth guiding channel. This allowed for integrated, continuous electrical stimulation, as well as the recording of cardiac tissues, and was observed to result in improved maturation of the cardiac tissue [109]. Whilst electrical sensing capabilities are traditionally embodied by 2D models, there is the ability of hydrogel scaffolds to be patterned on top of electrodes [110] or 3D electrodes [109]

Significant aspects of cardiac physiology and disease are replicated using organ-on-chip platforms, with cases involving hypertrophy (believed to be an increase in heart volume as a compensatory mechanism against overload) [111], fibrosis (commonly resulting in arrhythmia) [112], and ischaemia [113], all reported in the literature. According to Paloschi et al. [105], the vast majority of heart-on-chip devices reported in the literature state that cardiotoxicity is their primary goal. Perhaps rather than focusing on replacing conventional drug discovery automats with microfluidics, its potential lies in screening out discoveries prior to expensive animal and human trials taking place, stimulating in vivo-like conditions for better drug efficacy and toxicity predictions [114].

Heart-on-chip device development is very much still on-going and is a multidisciplinary field, where pharmacologists, engineers, biologists, regulators, and clinicians, among others, play a key role in the development of these models and, importantly, their respective validation [105]. Standardisation and harmonisation is key, not just for the technical aspects of organ-on-a-chip devices, but also in terms of the cells and tissues that are integrated into the chips [105]. Human cells are highly variable in terms of growth, stability, and function, particularly when primary tissues or stem cells are used as a source [105]. Users from the pharmaceutical sector consider access to human cell material, including patient material, to be a major challenge [115].

It has been suggested that the most effective single mechanism to propel microfluidic technologies to a mainstay in the discovery and development of new drugs is to codify a regulatory pathway specifically for organ-on-a-chip technologies [116].

### 6.2. Cardiac 3D Culture

As Zuppinger describes, 3D cardiac cultures can typically be split into the following two types:

- Scaffold matrix, typically hydrogel mixed and populated with a cell population and forming a strip between attachment sites, forming contracting myocardial tissue [117];
- Smaller cell aggregates, formed through cell self-assembly without matrix proteins [118,119,120].

Within cardiac muscle tissue, cardiac muscle cells typically cluster due to aggregation, alignment, concerted contractions, and some degree of cellular maturation. As Zuppinger et al. state, “actual embryonic development of a vascularized organ with chambers, working pump function and conduction system currently is not feasible to replicate in vitro” [52]. As Joddar et al. state, the overarching goal of cardiac organoid models is to establish a functional integration of cardiomyocytes with physiologically relevant cells, tissues, and ideally capillary-like networks, composed of endothelial cells [120]. Organoids can be both self-assembled and engineered with the support of biomaterials. Strategies that aim to accomplish such a feat include microfluidic technology-based approaches [107,121,122,123], microphysiological systems [124], microwells [125,126], microarray-based platforms [119], 3D bioprinted models [127,128], and electrospun fibre mat-based scaffolds [120,129,130]. A comparison of the advantages and disadvantages of scaffold-based models and cardiac spheroids is presented in Table 3.

Cardiac organoids have been developed with cardiomyocytes as the main cell types and have been described as functional tissue biomimicries [120,131]. Lewis-Isaraeli et al. self-assembled human heart organoids using human pluripotent stem cells and a three-step Wnt signalling modulation strategy, using chemical inhibitors and growth factors. Whilst they claimed the development of cardiac spheroids, it is important to note they reported that the tissue-matched human foetal cardiac tissues rather than mature tissue at the transcriptomic, structural, and cellular level [119]. They did, however, report that heart-like structures, in terms of structure, organisation, functionality, cardiac cell-type complexity, ECM composition, and vascularisation, were observed [119]. A range of other authors have developed self-assembled matrix-free cardiac spheroids for disease modelling in particular [132,133,134,135], and it has been suggested that the future of tissue engineering and disease modelling lies in assembly strategies, using cell spheroids (or organoids) as living building blocks to construct complex 3D tissue models, with spatial organisation [136]. It is worth noting, however, that for high-throughput drug development such complex time- and cell-intensive methodologies may prove unviable.

### 6.3. Materials for 3D Culture

Where scaffold-based models are applied, significant consideration must be given to the correct selection of materials. Stiffness and composition need to be individually adjusted to suit the requirements of certain cell types for their in vivo-like development and behaviour. In the field of stem cell research, Matrigel is often used to guide specific differentiation, but this may not be optimum for cardiac tissue formation [5]. Lemoine et al. reported successful 3D cultivation of iPSC-derived cardiomyocytes in a mixture comprising Matrigel, fibrin, and thrombin, as they suggested that this scaffold closely represented adult atrial and ventricular myocardium in terms of sodium currents [137]. However, other authors have identified collagen I as the most suitable 3D scaffold for mature rat cardiomyocytes, due to its superior cell-binding properties and facilitation of cellular migration [138].

Scaffold-based models of cardiac tissue have seen particular development for cardiac tissue engineering and their advantages for this area of research are without question. An extension of scaffold use for the formation of organoids is the process of bioprinting, namely the combination of a relevant cell source (often a cell line due to the significant number of cells required) and a bioink (for example, a fibrin-based composite hydrogel [127]) to create a cell-embedded, fully three-dimensional, physiologically relevant structure [127,139,140,141]. In one such study, by Wang et al., bioprinted cardiac tissue constructs were created with a spontaneous synchronous contraction in culture; they observed progressive cardiac tissue development and uniformly aligned, dense, and electromechanically coupled cardiac cells. These constructs exhibited physiologic responses to known cardiac drugs in terms of both beating frequency and contraction forces, as well as physiologically representative signalling mechanisms. In a review article on biomimetics in cardiac tissue engineering, the area of 3D bioprinting would have significant emphasis, indeed whole review articles have been written on the subject. However, this is a review on biomimetics in drug discovery and the limitations, as described in Table 2, with scaffold use for organoid development are further accentuated for larger scale bioprinting. A possible exception to this is application within ‘organ on a chip’, wherein the physiologically relevant structure can be combined with flow and continual nutrient delivery at a scale more viable for high-throughput culture.

Models are being developed that are able to provide biophysical stimulation of 3D tissues to model polygenic diseases over many months [79]. The complexity of such systems cannot be understated, combining, for example, microwells, conducting polymeric electrodes and myocardial tissues, created by combining CMs (ventricular, atrial, or both) and cardiac fibroblasts with hydrogel [79]. Such systems rely upon combined directed cell differentiation, along with electrical field conditioning, in order to engineer electrophysiologically distinct atrial and ventricular tissues [79]. Insights from such complex models of disease modelling are undoubtedly of value for both directing drug development and also provide the potential for the replacement or reduction in in vivo studies, but it is a field that is too vast and too complex to be considered here.

## 7. Future Challenges to the Implementation of More Biomimetic Approaches to Cardiac Drug Discovery

It is widely acknowledged that bioengineers have made major advancements in creating more physiological microenvironments compared to ‘highly unnatural tissue culture plastic’; however, challenges undoubtedly remain “in engineering an ECM that precisely recapitulates the structural intricacy, biochemical complexity and mechanical robustness of native cardiac ECM” [142]. The most advanced in vitro cardiac models rely upon induced pluripotent stem cells (iPSCs); however, the maturation of cardiomyocytes (CMs) has not yet been fully achieved and to get close to such a goal relies upon optimum mechanical and electrical stimulation [143,144,145,146]. These methodologies may be desirable for optimised cardiac tissue engineering for disease modelling and tissue regeneration, but such methodologies are costly, cell intensive, and time consuming, and are certainly not suited to the early stages of high-throughput drug screening. There are, additionally, concerns around standardisation and regulatory approval.

With a particular focus on biomimetic microfluidic devices, heart-on-a-chip models mostly feature ventricle-derived tissue, thereby limiting them to consideration of ventricular effects [5]. It has been hypothesised that additional knowledge of chamber-specific development and generation, as well as miniaturisation, is required to properly mimic the four-chamber structure, as well as electrophysiology, of the heart, ultimately presenting the possibility to reliably predict cardiotoxicity [5].

Organ-on-chip devices are certainly the most developed technologies and, in 2011, the president of the United States announced the establishment of the “Microphysiological System” research project [147]. The global organ-on-a-chip market was estimated at USD 54.6 million in 2021 and is expected to reach USD 697.7 million by 2028 [147], but they are still not routinely implemented within drug development pathways and do not have full regulatory approval. Wang et al. suggest that commercialisation will occur in a stepwise way and that it will be some time before such solutions are implemented into the early stages of the drug development pipeline for lead compound validation and optimisation. There is likely a compromise necessary between standardisation, reproducibility, and reliability in replicating the disease microenvironment and physiological drug response and proceeding towards patient-derived testing platforms for personalised precision medicine [148].

## Figures and Tables

**Table 1:** Overview of in vitro culture systems implemented for atherosclerosis drug discovery and screening. Modified from Chen et al. [30].

In Vitro Model	Features and Applications	Advantages	Challenges
Single-cell systems (2D)	Only one cell type in a tissue culture plate (TCP), evaluation of the drug and drug delivery systems	High availability, easy to use, low cost, high reproducibility, high throughput	Fails to mimic vascular structure or native plaque
Direct co-culture (2D)	Multi-cells cultured together on TCP, study on cell–cell interaction	Many of the advantages of single-cell systems, but media selection can be more problematic	Does not mimic native physiological structure, therefore it influences the nature of cell interactions with the extracellular matrix
Indirect transwell co-culture (2D)	Cells seeded in a TCP and a transwell insert, study of cellular responses via secretory pathways and cytokine production	Many of the advantages of single-cell systems, but media selection can be more problematic	Again, does not mimic native physiological structure
Cell sheet (2D)	Cells seeded on 2D scaffold or sheet of cells produced without scaffold presence, potential for therapeutic evaluation and investigation of influence on cell–cell interaction	Better at mimicking the vascular wall structure than other 2D culture methods, relatively easy to produce	Prone to spontaneous shrinkage, poor mechanical properties, more expensive than TCP-based systems
Microfluidic chip (2D)	Typically, endothelial cells are seeded onto a chip with flow systems, mechanistic studies, nanomedicine evaluation, allows real-time imaging	Microanalysis provides continuous monitoring and media supply. Dynamic culture, relatively easy to produce	Requires additional equipment, does not adequately model native 3D environment, costly
Spheroid (3D)	Cellular aggregates, provide 3D structure, mechanistic studies	Spherical structures more closely represent physiological cell–cell and cell ECM interactions	Limited capability or function compared to native tissue
Cell-laden hydrogel construct (3D)	Cells embedded within hydrogel scaffold, mechanistic studies	Scaffold provides ECM-mimicking environment, relatively easy to produce	Difficult to reproduce, poor mechanical properties
Tissue-engineered blood vessel (3D)	Reproduces native structure and size of the vessel, potentially with disease features, drug evaluation, mechanistic studies	Allows controlled stimuli (electrical, mechanical), dynamic culture, enables real-time imaging	Often not developed using arterial cells, typically lacks fibroblast layer, fails to induce advanced atherosclerotic plaque, expensive, time consuming and difficult to reproduce
Vessel on a chip (3D)	Micro-sized chip with vessel structure replicated, drug screening, mechanistic studies, real-time imaging	Allows controlled stimuli, partially vessel-like structure, dynamic culture, comparatively high throughput (certainly for 3D culture)	Expensive, lacks replication of multiple layers of the vessel, limited to drug screening, is not an actual sized vessel

**Table 2:** Pro and cons of small and large animal models of CVD. Recreated in full from Savoji et al., *Biomaterials* (2019) [2].

Pros and Cons of Animal Models of CVD	
Small Animal Models (Rodents)	Large Animal Models (Swine)
√ Easy breeding and handling	√ Closer to human anatomy, better tissue availability, and more accurate, minimally invasive measurements
√ Short reproductive cycle	√ Closer lipoprotein profile to humans
√ Relatively cheap	√ Moderately atherosclerosis sensitive on normal diet
√ Well-defined genome	√ Similar vascular lesion structure and lesion distribution to humans
√ Ease of genetic manipulation	√ Rare thrombosis due to plaque rupture
√ Large litter number	√ Suitable for translational research
× Resistance to atherosclerosis development in wild type	× Costly and difficult maintenance and handling
× Different gross anatomy compared to humans	× No genetic modifications
× Different lipoprotein profile to humans/high level of lipids	× Limited genetic models available
× Compromised lesion formation	× Rare thrombosis due to plaque rupture
× Absence of plaque rupture and thrombosis	× Ethical concerns

**Table 3:** A comparison of the advantages and disadvantages of scaffold-based models and cardiac spheroids. Modified with permission from Zuppinger et al. (2019) [52].

Features of Cardiac Spheroids	Advantage	Disadvantage
Small size of multi-cellular aggregates	Uses relatively small number of cells per data point	Methods such as protein chemistry and RNA extraction require pooling of spheroids
Made without additional scaffold proteins	No interference of scaffold proteins with microtissue development or assay outcome	ECM factors could improve survival and self-organisation of tissue
Assembles spontaneously due to gravity or a on non-adhesive surface	Spheroids quickly formed and ready for drug treatment and analysis	Little control over distribution of cell types, overall shape, or may result in multiple spheroids
Spheroids are floating in culture	Can be manipulated by pipetting and sedimentation, does not require manual handling	Spheroids may get lost or stuck on surfaces during pipetting steps
Show long-term spontaneous contractions	Motion activity and calcium cycling correlate with cell viability and drug treatment	No direct force measurement, non-linear cell alignment
Spheroids can be cultured in single wells	Miniaturised multi-well formats and compatible with plate readers	
**Feature of scaffold-based models (EHT)**	**Advantage**	**Disadvantage**
Shape is determined by scaffold/hydrogel mould	Shape can be tailored for application (screening, regeneration, maturation, regenerative medicine)	Uses large number of cells per tissue
Made with scaffold biomaterial and ECM proteins	Hydrogel can be adapted for different organotypic functions and pathologies	Limited diffusion, risk of breaking, unequal cell distribution, potential interference with assays
Tissues attached to support structures	Sensors can be integrated	Manual steps necessary, small number of tissues from the same batch of cells
Linear alignment of muscle cells	Mechanical and electrical training possible, physiological function in disease models, force assessment	
Training protocols show improved maturation of HiPSC-CM	Technology development particularly towards tissue engineering applications	
